# Revisiting nitrates use in pre-shock state of contemporary cardiogenic shock classification

**DOI:** 10.3389/fcvm.2023.1173168

**Published:** 2024-01-04

**Authors:** Rasha Kaddoura, Ashfaq Patel, Abdul Rahman Arabi

**Affiliations:** ^1^ Pharmacy Department, Heart Hospital, Hamad Medical Corporation, Doha, Qatar; ^2^ Department of Cardiology, Heart Hospital, Hamad Medical Corporation, Doha, Qatar

**Keywords:** cardiogenic shock, isosorbide dinitrate, pre-shock, nitrates, nitroglycerine, SCAI, vasodilators

## Abstract

Patients at each shock stage may behave and present differently with a spectrum of shock severity and adverse outcomes. Shock severity, shock aetiology, and several factors should be integrated in management decision-making. Although the contemporary shock stages classification provided a standardized shock severity assessment, individual agents or management strategy has not yet been studied in the context of each shock stage. The pre-shock state may comprise a wide range of presentations. Nitrate therapy has potential benefit in myocardial infarction and acute heart failure. Herein, this review aims to discuss the potential use of nitrate therapy in the context of the pre-shock state or stage B of the contemporary shock classification given its various presentations.

## Introduction

Patients presenting with cardiogenic shock (CS) are a heterogenous population (1) in terms of presentations, therapeutic benefit, outcomes, and prognosis based on existing comorbidities and CS aetiology, phenotypes, and severity (2–4). Furthermore, defining CS, independently from shock severity, maybe equally challenging (5). Thus, the Society for Cardiovascular Angiography and Interventions (SCAI) introduced a consensus-based risk stratification for CS in five stages (A to E) in 2019 (2) that was endorsed by various international societies (4), widely adopted by clinicians, and validated across the CS spectrum by field experts. The SCAI classification has then been updated after detailed revision of the validation studies to help refining the classification scheme and accommodating variabilities in clinical parameters of patient presentation. SCAI shock classification may allow a uniform shock severity assessment that is an important element of management and prognostication for CS patients. However, patient in each SCAI stage may behave distinctly and may present with a range of disease severity and risk of mortality. Although hemodynamic parameters are generally used for CS diagnosis, a formal definition for each hemodynamic shock phenotype that may precisely guide therapy is absent. Other elements to integrate in decision-making for CS patients include shock aetiology, congestion severity, ventricular involvement, presence of organ failure, other types of shock states, and additional risk factors and comorbidities (5).

Individual agents or management strategy has not yet been studied in the context of each SCAI shock stage. The pre-shock state or SCAI stage B as defined by the SCAI shock classification may comprise a wide range of presentations including those related to patients with acute heart failure and myocardial infarction. Nitrate therapy has potential benefit in myocardial infarction and acute heart failure. Collectively, available evidence from nitrates studies demonstrated favourable hemodynamic effects and symptomatic improvement (6, 7). Herein, an electronic PubMed literature search was conducted for this review that aims to discuss the potential use of nitrate therapy in the context of the pre-shock state or stage B of the contemporary SCAI shock classification given its various presentations such as patients with pulmonary edema, heart failure either *de novo* or acute-on-chronic, or myocardial infarction complicated with CS.

## SCAI shock classification

**Figure 1:**
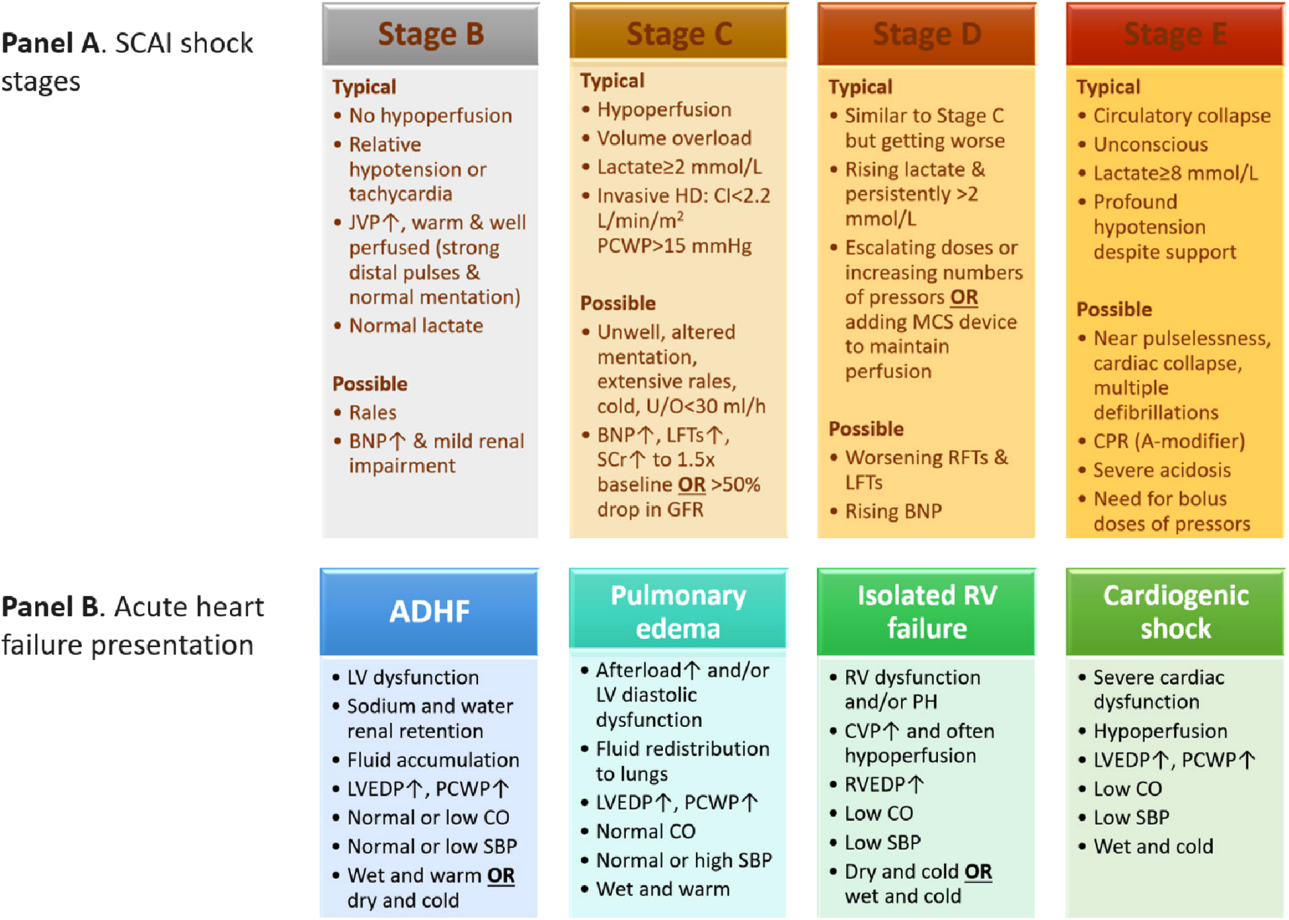
Characteristics of SCAI shock stages (panel A) (5) and acute heart failure presentation phenotypes (panel B) (22). ADHF, acute decompensated heart failure; BNP, brain natriuretic peptide; CI, cardiac index; CO, cardiac output; CPR, cardiopulmonary resuscitation; CVP, central venous pressure; GFR, glomerular filtration rate; HD, hemodynamics; JVP, jugular venous pressure; LFTs, liver function tests; LV, left ventricular; LVEDP, left ventricular end-diastolic pressure; MCS, mechanical circulatory support; PCWP, pulmonary capillary wedge pressure; RFTs, renal function tests; RV, right ventricular; RVEDP, right ventricular end-diastolic pressure; SBP, blood pressure; SCAI, Society for Cardiovascular Angiography and Interventions; SCr, serum creatinine; U/O, urine output.

The SCAI scheme for CS comprises the following stages: stage A or at-risk, stage B or beginning or pre-shock state, stage C or classic, stage D or deteriorating, and stage E or extremis. Each stage is described by physical bedside exam findings, hemodynamic parameters, and biochemical markers (Figure 1, Panel A) (5). Moreover, when the (A) modifier is integrated in each CS stage, it can provide a prognostic mean to identify patients at risk of cardiac arrest or poor outcomes (2). The re-assessment of SCAI stages at various intervals after patient presentation has been suggested to provide further guidance on prognosis and treatment options (e.g., escalating or deescalating therapy). As such, an improved SCAI stage by one category was a positive prognostic marker and vice versa (5). Pharmacological and non-pharmacological (i.e., mechanical) circulatory support are usually needed to combat hypotension and restore tissue hypoperfusion (8). In stage C there is hypoperfusion that usually requires vasoactive agents or mechanical circulatory support. In stage D, the initial supportive measures and interventions fail, which may progress to stage E. The latter represents refractory shock with impending or actual circulatory collapse regardless of the escalated level of supportive measures (5). The less severe stages A and B may not necessitate circulatory support since the tissue perfusion is preserved. In stage A patients are usually stable with an acute cardiac presentation that puts them at risk to develop CS. Stage B or pre-shock state includes patients with preserved systemic tissue perfusion but with signs of hemodynamic instability such as relative hypotension and compensatory tachycardia, or with abnormal hemodynamic parameters measured invasively such as low cardiac output (5). The characteristics of patients presenting with stage B from the SCAI classification validation studies are discussed below.

## Pre-shock state in validation studies

Since the introduction of the SCAI stages in 2019, the SCAI classification has been validated by several studies. The studies, ranged from 166 to 10,004 participants, found an association between SCAI stages and mortality risk in various settings (9–17), i.e., higher SCAI stage was correlated with higher mortality rate, both at short- and long-term follow-up (9, 11, 14). Five studies focused on CS with or without myocardial infarction (9, 10, 15–17), three studies included patients in cardiac intensive care units (11–13), and one study recruited patients with out of hospital cardiac arrest (14). The prevalence, definition criteria, and outcomes of the SCAI classification stages have varied between the validation studies (5). For example two studies did not use specific criteria to assess the CS stage (9, 10), while five studies used study specific SCAI stage criteria (11, 13, 16). The prevalence, definition, and variables used for SCAI stage B in the validation studies are presented in Table 1. Overall, there was some variations with regards the use of vasopressors especially in SCAI stages B and C (5). The distinction between the pre-shock state and the classic CS as the unchanged and reduced perfusion states, is important because hypoperfusion places patients at increased risk of death in comparison with those with unchanged perfusion. Thus, this requires involving various clinical and laboratory information (18). Laboratory biomarkers alone may be insufficient. It has been suggested that lactate, as a marker of hypoperfusion, of a level above 2 mmol/L may reflect at least CS SCAI stage C. However, some patients with normal lactate level may have signs of tissue hypoperfusion or not related to hemodynamic such as in patients with chronic heart failure and reduced cardiac index. On the other hand, other causes than shock can lead to elevated serum lactate level such as compartment syndrome (5).

**Table 1:** Definition of SCAI stage B and variables used in validation studies.

Author recruitment period study design	Prevalence of stage B population	Definition of stage B	Variable used in SCAI stages
Baran et al. (9) 2019–2020 Prospective Single center	10/166 (6%) CS patients	As per SCAI clinical expert consensus statement on the classification of CS (2)	As per SCAI clinical expert consensus statement (2)
Hanson et al. (10) 2016–2019 Prospective Multicenter	0/300 Patients with CS from acute MI	Physical exam: elevated JVP; rales; strong distal pulses; normal mentation Biochemical markers: normal lactate; minimal renal function impairment; elevated BNP Hemodynamics: SBP <90 or MAP <60 or >30 mmHg drop; pulse ≥100; CI ≥2.2; PA saturation ≥65%	Vital signs Lactate CPR Vasoactive drugs MCS
Jentzer et al. (11) Retrospective Single centre 2007–2015	2,998/10,004 (30%) CICU patients	Patients meeting all the following: 1. One or more criterion for hypotension and/or tachycardia during first 1 h after admission: a. Minimum SBP <90 mmHg b. Minimum MBP <60 mmHg c. Maximum HR >100 bpm d. Admission HR > admission SBP e. Mean HR > mean SBP 2. No criteria for hypoperfusion: a. admission lactate ≤2 mmol/L; b. 24-hour urine output ≥720 ml; c. 24-hour creatinine increase <0.3 mg/dl	Vital signs Lactate Renal function Vasoactive drugs MCS
Jentzer et al. (12)	2,786/9,096 (30.6%) CICU survivors	Definition as per Jentzer et al. 2019 (above) (11)	As per Jentzer et al. (11)
Lawler et al. (13) Retrospective Multicenter 2017–2019	138/1,991 (7%) CICU or CS patients	ACS or HF meeting all the following: 1. Either of the following criteria for hypotension: a. SBP <90 mmHg for ≥30 min b. Need for vasopressors/inotropes to maintain SBP ≥90 mmHg 2. GFR ≥60 m/min 3. 3. Normal lactate <2 mmol/L	Diagnosis of CS Lactate pH LFTs Renal function Vasoactive drugs MCS
Pareek et al. (14) 2012–2017 Retrospective Single centre	94/393 (23.9%) Patients with OHCA	OHCA patient meeting all the following: (Without hypoperfusion) 1. Either of the following criteria for hypotension and/or tachycardia: a. SBP >90 mmHg and HR >100 bpm; b. Low-dose bolus vasopressor to maintain SBP >90 mmHg 2. GFR >60 ml/min	Vital signs Vasoactive drugs
Schrage et al. (15) 2009–2017 Retrospective Single centre	35/1,007 (3.5%)' Patients with CS or large MI	Patients having clinical evidence of relative hypotension or tachycardia, but without hypoperfusion. Signs/symptoms of CS or large MI with HF > SBP (ratio >1) plus all the following: a. No vasoactive drugs use; b. Arterial lactate <2 mmol/L (or venous lactate <2.5 mmol/L) i.e., no hypoperfusion	Diagnosis of CS Vital signs CPR Lactate Vasoactive drugs
Thayer et al. (16) 2016–2017 Prospective Multicenter	46/1,414 (3.3%) CS Patients	CS patients are those exhibiting early symptoms: If lactate NOT available: CS patients NOT receiving any vasoactive drugs or MCS devices. If lactate available: CS patient meeting all the following: a. No vasoactive drugs or MCS devices; b. Lactate <2 mmol/L	Lactate Vasoactive drugs MCS

ACS, acute coronary syndrome; BNP, B-type natriuretic peptide; bpm, beat per minute; CI, cardiac index; CICU, cardiac intensive care unit; CPR, cardiopulmonary resuscitation; CS, cardiogenic shock; GFR, glomerular filtration rate; HF, heart failure; HR, heart rate; JVP, jugular venous pressure; LFTs, liver function tests; MAP, mean arterial pressure; MCS, mechanical circulatory support; MI, myocardial infarction; OHCA, out-of-hospital cardiac arrest; PA, pulmonary artery; SBP, systolic blood pressure; SCAI, Society for Cardiovascular Angiography and Interventions.

## Description and presentation of patients in pre-shock state

Patients presenting with SCAI stage B are usually described to have signs and symptoms of hemodynamic instability such as relative hypotension or tachycardia in the absence of hypoperfusion. Bedside findings show warm and well-perfused patients with strong distal pulsation and normal mentation but typically with elevated jugular venous pressure and infrequent rales in the lung fields. Lactate levels are typically normal with possibly elevated B-type natriuretic peptide or minimal acute renal impairment. Hemodynamically, they usually have relative tachycardia and hypotension (5). Characteristics and clinical outcomes of patients in SCAI stage B reported in the validation studies are summarised in Table 2. When we pooled the variables from the validation studies, we found that patients in the pre-shock state had a mean age of 66 years, 67.6% of patients were males, and 22.4% were smokers. The most common background comorbidities were coronary artery disease (57.8%), hypertension (55.2%), heart failure (44.8%), diabetes (28.4%), myocardial infarction (21.5%), renal impairment (19.8%) and stroke (12.2%) (Table 2).

**Table 2:** Baseline characteristics and outcomes of patients in SCAI stage B reported in validation studies.

Variable	Baran et al. (9)	Jentzer et al. (11)	Lawler et al. (13)	Pareek et al. (14)	Schrage et al. (15)	Thayer et al. (16)	Pooled variable Mean ± SD or % (95% CI)
	(*n* = 10)	(*n* = 2,998)	(*n* = 138)	(*n* = 94)	(*n* = 35)	(*n* = 46)	
Demographics							
Age (years)	44.2 ± 15.9	66.4 ± 15.7	66 (56–74)^a^	61.1 ± 24	72 ± 16	54.6 ± 16	66.03 ± 15.50 (*n* = 3,321)
Male gender	6/10 (60%)	1,782/2,998 (59.4%)	85/138 (61.6%)	75/94 (79.8%)	26/35 (74.3%)	33/46 (71.7%)	67.61% (59.34–75.37)
							(2,007/3,321)
Smoking (active)	1/10 (11.1%)	–	25/138 (18.2%)	–	12/35 (34.3%)	–	22.46% (11.99–35.08)
							(38/183)
Comorbidities							
Hypertension	8/10 (80%)	–	77/138 (55.8%)	–	24/35 (68.6%)	12/46 (26.1%)	55.25 (35.57–74.11)
							(121/229)
Diabetes	3/10 (30%)	851/2,992 (28.4%)	39/138 (28.3%)	–	12/35 (34.3%)	11/46 (23.9%)	28.46 (26.91–30.05)
							(916/3,221)
Dyslipidemia	–	–	–	–	8/35 (22.9%)	–	8/35 (22.9%)
							One study
CVA/TIA	–	346/2,777 (12.5%) (12)	10/138 (7.2%)	–	6/35 (17.1%)	4/46 (8.7%)	12.23 (11.08–13.46)
							(366/2,996)
Coronary artery disease	–	–	51/138 (37%)	60/77 (77.9%)	–	–	57.85 (18.89–91.71)
							(111/215)
Myocardial infarction	–	581/2,992 (19.4%)	–	–	10/35 (28.6%)	–	21.59 (14.35–29.86)
							(591/3,027)
Heart failure	8/10 (80%)	638/2,992 (21.3%)	65/138 (47.1%)	–	–	–	44.80 (20.70–70.28)
							(711/3,140)
Renal impairment	–	604/2,992 (20.2%)	19/138 (13.8%)	–	7/35 (20%)	14/46 (30.4%)	19.89 (15.08–25.18)
							(644/3,211)
Admission diagnosis/Shock cause							
ACS/Ischemic CS	–	1,172/2,968 (39.4%)	–	56/94 (59.6%) (STE)	24/35 (68.6%)	2/46 (4.4%)	40.58 (20.80–62.11)
							(1,254/3,143)
Heart failure	–	1,562/2,968 (52.5%)	–	–	11/35 (31.4%)	40/46 (87%)	58.22 (32.83–81.47)
							(1,613/3,049)
Cardiac arrest	1/10 (10%)	311/2,968 (10.5%)	12/138 (8.7%)	–	–	–	10.43 (9.38–11.52)
							(324/3,116)
Shockable rhythm	–	–	–	73/94 (77.7%)	–	–	73/94 (77.7%)
							One study
AF/SVT	–	1,150/2,968 (38.7%)	–	–	–	–	1,150/2,968 (38.7%)
							One study
VT/VF	–	516/2,968 (17.4%)	–	–	–	–	516/2,968 (17.4%)
							One study
CPR	–	–	–	–	9/35 (25.7%)	–	9/35 (25.7%) One study
CPR duration (min)	–	–	–	–	6.2 ± 5.1 (*n* = 35)	–	6.2 ± 5.1 (*n* = 35)
Severity of illness							
APACHE-III score	–	60.8 ± 20.4	–	–	–	–	60.8 ± 20.4 (*n* = 2,998)
							One study
APACHE-IV (mortality)	–	15.8 ± 16.7	–	–	–	–	15.8 ± 16.7 (*n* = 2,998)
							One study
SOFA score (Day 1)	–	3.4 ± 2.7	4 (3–6)^a^	–	–	–	3.43 ± 2.62 (*n* = 3,136)
Severe AKI	–	333/2,674 (12.4%)	–	–	–	–	333/2,674 (12.4%)
							One study
Late deterioration	–	190/2,998 (6.3%)	–	–	–	–	190/2,998 (6.3%) One study
Vital signs/Hemodynamics							
Systolic BP (mmHg)	106 (101–147)^a^	114.4 ± 26.1 (*n* = 2,980)	–	118.3 ± 28.2	127.4 ± 29.6	–	114.66 ± 26.18 (*n* = 3,119)
Diastolic BP (mmHg)	69.5 (60.8–91)^a^	67.1 ± 18.6 (*n* = 2,884)	–	70.4 ± 19.7	76.3 ± 21.5	–	67.32 ± 18.63 (*n* = 3,023)
MAP (mmHg)	81 (73.3–114.3)^a^	79.6 ± 19.6 (*n* = 2,884)	–	–	–	71.8 ± 7.5	79.50 ± 19.39 (*n* = 2,940)
Heart rate (bpm)	104.7 ± 25.7	93.1 ± 26.8 (*n* = 2,980)	–	96.9 ± 22.6	90.1 ± 24.5	75.2 ± 12.8	92.95 ± 26.45 (*n* = 3,165)
Respiratory rate (bpm)	–	19.1 ± 6.0 (*n* = 2,875)	–	–	–	–	19.1 ± 6.0 (*n* = 2,875) One study
Urine output (liter)	–	2.26 ± 1.42 (First 24 h) (*n* = 2,908)	–	–	–	–	2.26 ± 1.42 (First 24 h) (*n* = 2,908) One study
Cardiac index (L/min/m^2^)	2.3 ± 0.9 (Fick) 3.18 ± 1.6 (TD)	–	–	–	–	1.9 ± 0.3 (*n* = 45)	1.97 ± 0.4 (*n* = 55)
Cardiac output (L/min)	4.7 ± 2.4 (Fick)	–	–	–	–	3.8 ± 0.7 (*n* = 45)	3.96 ± 0.98 (*n* = 55)
	6.51 ± 3.9 (TD)						
CPO (W)	0.77 ± 0.41 (Fick)	–	–	–	–	0.6 ± 0.1 (*n* = 45)	0.63 ± 0.15 (*n* = 55)
PCWP (mmHg)	23.3 ± 6.5	–	–	–	–	16.5 ± 7.3 (*n* = 45)	17.71 ± 7.16 (*n* = 55)
PAP (mmHg)	–	–	–	–	–	27 ± 11.3 (*n* = 44)	27 ± 11.3 (*n* = 44) One study
Ejection fraction	31.3 ± 23.4%	–	–	42.8 ± 9.7%	–	65% (*n* = 1)	41.91 ± 10.90 (*n* = 105)
Laboratory tests							
Lactate (mmol/L)	2.5 (1.2–13.5)^a^	1.3 ± 0.4 (*n* = 638)	–	4.62 ± 3.35	1.5 ± 0.5	1.4 ± 0 (*n* = 1)	1.75 ± 0.79 (*n* = 778)
pH	–	7.36 ± 0.1 (*n* = 968)	–	7.25 ± 0.13	7.37 ± 0.07	7.4 ± 0.1 (*n* = 2)	7.35 ± 0.10 (*n* = 1,099)
Anion gap (mEq/L)	–	11.5 ± 3.2 (*n* = 2,677)	–	–	–	–	11.5 ± 3.2 (*n* = 2,677) One study
Bicarbonate (mEq/L)	–	23.9 ± 4.4 (*n* = 2,905)	–	–	–	25.7 ± 3 (*n* = 44)	23.92 ± 4.37 (*n* = 2,949)
Hemoglobin (g/l)	10.6 ± 2.5	11.9 ± 2.2 (*n* = 2,890)	–	–	–	–	11.89 ± 2.20 (*n* = 2,900)
TnT (mg/dl)	0.3 (0.23–0.58)^a^	1.7 ± 3.2 (Peak) (*n* = 1,897)	–	–	–	–	1.69 ± 3.18 (*n* = 1,907)
TnI (ng/L)	–	–	–	–	1,265 ± 2,039 (hs)	–	1,265 ± 2,039 (*n* = 35)
							One study
BNP	–	–	–	–	–	–	–
ALT (U/l)	–	76.2 ± 222.2 (*n* = 1,897)	–	–	–	–	76.2 ± 222.2 (*n* = 1,897)
							One study
AST (U/L)	24 (8–109)^a^	–	–	–	180 ± 307	32 ± 19.9 (*n* = 37)	96.29 ± 144.5 (*n* = 82)
BUN (mg/dl)	–	27.0 ± 18.9 (*n* = 2,878)	–	–	–	28.6 ± 16.6	27.00 ± 18.89 (*n* = 2,924)
Serum creatinine (mg/dl)	2.8 ± 4.1	1.3 ± 1.0 (*n* = 2,887)	–	1.07 ± 0.4	–	1.3 ± 0.4	1.29 ± 0.98 (*n* = 3,037)
GFR (ml/min)	43.3 ± 19.1	–	–	–	48.5 ± 21.1	–	47.34 ± 20.68 (*n* = 45)
Therapies/procedures							
Vasoactive agents use	7/10 (70%)	11.3% (First 1 h)	0	–	0	0	–
Number of vasoactive agents	Initial: 1 (0–1.25)	0.1 ± 0.4 (First 1 h)	0	–	–	0	0.10 ± 0.39 (*n* = 3,008)
	Mean: 0.81 ± 0.41						
	24 h: 0.5 (0–1.25)	–	–	–	–	–	–
	Mean: 0.56 ± 0.41						
Agent(s) used	E, D, M, NE, V	–	–	–	–	–	–
MCS	30%	12.1%	5%	28.7%	–	0	–
IABP	20%	11%	3.6%	27.6%	–	0	–
Impella®	0	0.2%	–	0	8.6%	0	–
ECMO	10%	0.9%	–	1.1%	2.9%	0	–
Coronary angiogram	–	49.1%	–	81.9%	–	–	–
PCI	–	31.1%	–	66.2%	–	–	–
Intubation	30%	–	–	–	25.7%	–	–
Mechanical ventilation	–	14%	18.1%	–	–	–	–
Dialysis/RRT	–	4.6%	0	14.9%	–	–	–
RBC transfusion	–	13.4%	–	–	–	–	–
Invasive monitoring/PAC	–	7.9% (12)	37%	–	–	–	–
In-hospital mortality	0	∼6% Adjusted OR 1.53 (against stage A)	2.2%	–	–	0	–
CICU mortality	0	∼3%	1.5%	–	–	–	–
30-day mortality	0	∼3% (12)	–	33% OR 1.21 (95% CI 0.66–2.20)	–	–	–
12-month mortality	–	∼43% (12)	–	35.1%	–	–	–
Poor neurological outcome (CPC 3–5)	–	–	–	42.6% (at 12-month)	–	–	–
30-day survival	100%	–	–		66.1% (95% CI 50.2–87) OR 0.43 (95% CI 0.19–0.92, *P *= 0.36) (against stage C)	–	–
One-year survival	100%	81.6% (12)	–	–	–	–	–
30-day hospital readmission	∼11% (12)	–	–	–	–	–	–

95% CI, confidence interval; ACS, acute coronary syndrome; AF, atrial fibrillation; AKI, acute kidney injury; ALT, alanine transaminase; APACHE, Acute Physiology and Chronic Health Evaluation; AST, aspartate-aminotransferase; BP, blood pressure; bpm, beats or breaths per minute; BNP, brain natriuretic peptide; BUN, blood urea nitrogen; CICU, cardiac intensive care unit; CPC, Cerebral Performance Category; CPO, cardiac power output; CPR, cardiopulmonary resuscitation; CS, cardiogenic shock; CVA, cerebrovascular accident; D, dobutamine; E, epinephrine; ECMO, extracorporeal membrane oxygenation; GFR, glomerular filtration rate; hs, high sensitivity; IABP, intra-aortic balloon pump; M, milrinone; MAP, mean arterial pressure; MCS, mechanical circulatory support; MD, mean difference; SD, standard deviation; min, minute(s); NE, norepinephrine; OR, odds ratio; PAC, pulmonary artery catheter; PCI, percutaneous coronary intervention; PAP, pulmonary artery pressure; PCI, percutaneous coronary intervention; PCWP, pulmonary capillary wedge pressure; RBC, red blood cell; RRT, renal replacement therapy; SD, standard deviation; SOFA, Sequential Organ Failure Assessment; STE, ST-segment elevation; SVT, supraventricular tachycardia; TD, thermodilution; TIA, transient ischemic attack; TnI/TnT, cardiac troponin; V, vasopressin; VF, ventricular fibrillation; VT, ventricular tachycardia.

^a^ Converted to mean ± standard deviation.

Patients in pre-shock state may present with pulmonary edema, acute heart failure either as *de novo* or acute-on-chronic, or myocardial infarction complicated with CS, therefore, shock aetiology can impact initial presentation and outcomes (5). Acute coronary syndrome (ACS) may precipitate 32% of acute heart failure cases and more patients are likely to present with *de novo* acute heart failure, i.e., 61% of the cases. Patients presenting with acute heart failure and ACS are significantly more likely to experience CS and pulmonary edema in comparison with their counterparts presenting without ACS, although heart rate, blood pressure and biochemistry tests on admission did not differ between the comparison groups. Initial treatment differed significantly between the two groups, patients with ACS received more intravenous medications (opioids, diuretics, nitrates, vasopressors, and inotropes) and coronary revascularization procedures. Although long-term survival at five years did not differ between the groups, death at 30 days was significantly higher in patients presenting with ACS (adjusted odds ratio (OR) 2; 95% confidence interval (CI): 1.07–3.79, *p* = 0.03) (19). In comparison with acute heart failure but in the absence of ACS, patients with acute decompensation on top of chronic heart failure may have different symptoms and hemodynamic parameters upon presentation and they may be able to tolerate lower blood pressure and cardiac output (20), i.e., due to adaptations and compensatory mechanisms. Thus, chronic heart failure patients may acutely present with a lower SCAI stage which may give false reassurance despite their high-risk hemodynamic parameters (21). As a result, physical findings and hemodynamic parameters should be interpreted within the clinical context. The later SCAI C, D, and E stages may appear similar irrespective of the underlying chronicity, whereas in SCAI A and B stages the differences in physical and hemodynamic findings can be more evident (Figure 1, Panel A) (5). Our pooled variables from the validation studies showed that the cause of shock or diagnosis at admission was heart failure, ACS, or cardiac arrest in 58.2%, 40.5%, or 10.4% of patients, respectively. The mean systolic, diastolic, and mean blood pressure values were 114.6, 67.3, and 79.3 mmHg, respectively with a mean heart rate of 92.9 beats per minute (bpm). The mean cardiac index was 1.97 L/min/m^2^, cardiac output was 3.96 L/min, cardiac power output (CPO) was 0.63 W, pulmonary capillary wedge pressure (PCWP) was 17.7 mmHg, and ejection fraction was 41.9%. Lactate level and pH were 1.75 mmol/L and 7.35, respectively.

## Pre-shock state in the context of acute heart failure guidelines

Acute heart failure is a heterogenous condition in which management is decided based on clinical presentation and starts with identifying the underlying cause. The 2021 European guidelines characterized acute heart failure by four main clinical presentations, based on congestion signs and/or peripheral hypoperfusion, with probable overlap between them. The clinical presentations comprise acute decompensated heart failure (ADHF), acute pulmonary edema, isolated right ventricular failure, and cardiogenic shock. Figure 1 (Panel B) summarizes the four clinical presentations of acute heart failure (22). ADHF is considered the most common among the four clinical presentation phenotypes of heart failure (50%–70%), and often occurs in patients with underlying heart failure and left ventricular dysfunction but may also include right ventricular dysfunction. Patients often present with fluid overload and signs of increased intraventricular pressure. Distinct from ADHF phenotype, acute pulmonary edema has more rapid onset (i.e., hours vs. days) and the main alteration is fluid redistribution to the lungs and the resultant acute respiratory failure (22). Patients with ADHF or acute pulmonary edema share various characteristics with patients presenting with SCAI stage B or pre-shock state such as the absence of tissue hypoperfusion, relative hypotension, possible rales, and being warm and well perfused, etc.

## Nitrates therapy

### Efficacy and safety of nitrate therapy

Organic nitrates (e.g., nitroglycerin, isosorbide dinitrate, and isosorbide mononitrate) release nitric oxide through an enzymatic process unlike sodium nitroprusside that releases nitric oxide spontaneously. Nitric oxide eventually causes smooth muscle relaxation and vasodilatation (23). Intravenous nitrates and nitroprusside reduce preload and afterload through dilating both venous and arterial vessels. Nitrates are more powerful on peripheral veins, whereas nitroprusside produces a balanced venous and arterial dilation (22). Organic nitrates at low doses cause venous dilatation, whereas arteries and coronary arteries dilate at higher doses. By provoking venous and arterial dilation, intravenous nitrates can reduce the increased left ventricular filling pressures and systemic vascular resistance without affecting tissue perfusion (23), and improve stroke volume and cardiac output (24). As a result, nitrates provide marked improvement in acute pulmonary edema in which there is rapid deterioration especially in patients who have an acute rise in systemic vascular resistance and left ventricular filling pressures due to decreased baseline diastolic and systolic reserve (23). In addition, nitrates are effective agents in relieving pulmonary congestion and chest pain in patients presenting with acute coronary syndrome and heart failure because they are powerful venous vasodilators and anti-ischemic drugs (25).

Intravenous nitrates at higher doses dilates coronary arteries and enhances collateral blood flow which is desirable in ischemia but the subsequent tachyphylaxis usually necessitates dose escalation (7), due to the attenuation of the favorable hemodynamic effects (23). This pseudo- or early tolerance seems to be induced by counter-regulatory responses of neurohormone such as increased vasopressin, noradrenaline, and renin activity which lead to plasma volume increase due to sodium and water retention. A true tolerance can result from continued nitrate administration leading to changes in smooth muscle and endothelial functions (23). Other drawbacks of nitrates’ use include headache, hypotension, dizziness, and free radical production (7, 26). The rates of reported adverse events differed among studies and disease states. With nitrate use, headache was reported in 2%–26% of patients with acute myocardial infarction and in 12% of those with acute heart failure. Furthermore, the incidence of hypotension and dizziness was 1%–48% and 1% in acute myocardial infarction and 5%–10% and up to 29% in acute heart failure, respectively (25). Nitrates should not be used in patients with hypotension, chronic obstructive pulmonary disease (i.e., acute heart failure mimics), and left ventricular outflow tract obstruction because vasodilation does not provide benefit in these conditions. In conditions with vascular obstruction such as pulmonary embolism, nitrates can cause excessive hypotension and cautious use should be considered in preload-dependent patients. Nitrates should not be used concurrently with phosphodiesterase inhibitors (e.g., sildenafil, tadalafil) (27).

### Nitrate therapy in myocardial infarction

In addition to the anti-anginal effect due to multiple mechanisms, nitrates decrease ventricular dilatation in acute myocardial infarction which help improving mitral regurgitation and pulmonary congestion (28). Nitrates have also reduced myocardial infarct size or its expansion and improved global or regional left ventricular function (6). Very early small reports that studied both oral and intravenous nitrates in acute myocardial infarction showed a trend towards reduced reinfarction and mortality (28), as was shown in a pooled analysis published in 1988. The analysis included 10 small randomised controlled trials (*n* = 2,000) using intravenous nitroglycerin (seven studies) or nitroprusside (three studies). Both vasodilators decreased mortality and the reduction was the greatest at short term follow-up especially in the first week, with non-significant reductions after the early period (29). A subsequent review analysed the seven intravenous nitrate studies (*n* = 850) then analysed them with additional studies that used oral nitrates. Intravenous nitrates reduced the odds of death by 48% (95% CI: 25–64, *p* < 0.001), a benefit that was not demonstrated with the oral nitrates, but combining all nitrates studies reduced the odds of death by 32% (95% CI: 12–47, *p* < 0.001). However, the conclusion was limited by small-scale studies (6). Then the two large, randomised trials, ISIS-4 (30) and GISSI-3 (31) which administered nitrates within 24 h of myocardial infarction onset, refuted the mortality benefit. The divergent results were justified by the possible lower nitrate doses used and the widespread use of nitrates in the control groups that could have diluted the beneficial effects (28).

### Nitrate therapy in heart failure and pulmonary edema

In acute decompensated heart failure, there is reduced nitric oxide bioavailability hence exogenous nitrates are needed (26). Furthermore, patients with acute heart failure usually present with elevated left ventricular filling pressure and normal or high blood pressure. In this condition, vasodilators improve symptoms and hemodynamic parameters. They are frequently used with loop diuretics with much of their acute effect is suggested to be due to venodilation (7). Intravenous nitroglycerin, the most used vasodilator, has fast onset and offset of action with an expected dose-response effects on both peripheral circulation and overall hemodynamic parameters. It decreases left and right ventricular filling pressures and the afterload (26). Nitrates have been used in acute heart failure for many years (32), but the evidence is limited (33, 34) and mostly evaluated hemodynamic rather than clinical outcomes in small cohorts of patients (33). As a result, their administration substantially varied between patients (6%–70%) (23, 34) and nitrate use has been less standardised in clinical practice (23). Very early studies on nitrates use in heart failure were of small size and found improved exercise capacity without reliable mortality data (6). A Cochrane review included four randomised trials (*n* = 634) that compared nitrates with any non-nitrate comparator in patients with acute heart failure syndromes with or without myocardial infarction. There was not significant difference in symptomatic relief and hemodynamic parameters between the comparison groups. However, study designs and enrolled patients were heterogenous, and the trials were of low quality (32). The analysis of the 3COP randomised trial in patients with acute cardiogenic pulmonary edema demonstrated that intravenous nitrates did not reduce mortality rate (35).

## Challenges with nitrate therapy

### Underutilization of nitrate therapy

Several studies have suggested that despite its benefit, nitrate therapy is underutilized in the clinical practice (27). It has been reported that only 12% of patients who were suitable for intravenous nitrate therapy received it. Those patients were more likely to have hypertension or myocardial ischemia (24). Another study reported 42% of patients with acute heart failure received nitrates who often had pulmonary edema or hypertension (36). Semi-structured interviews with 40 hospital physicians in the United Kingdom found that intravenous nitrates were considered in 37% of clinical decisions in treating virtual acute heart failure patients with noticeable variability between the physicians. Physicians’ beliefs and perceptions were found to heavily influence their decisions (37). Hypotension is probably the most prominent property that limits the use of nitrates, due to the potential end-organ tissue perfusion. For example, in patients with acute heart failure and reduced cardiac reserve, nitrates may steeply lower the blood pressure leading to hemodynamic instability, renal failure, ischemia, and possible over shock, all of which are associated with increased risk of mortality. In acute heart failure, there is no consensus on the optimal dosing regimen for nitrate therapy and the published studies have based nitrate dose up-titration on pre-specified blood pressure limits and physician's clinical judgement (23). Hence, there is inconsistency between the international guidelines recommendations with regards the routine use of nitrates in acute heart failure, which was attributed to the absence of robust evidence (24). The general approach is to use nitrate therapy when blood pressure is 110 mmHg or above, and to be avoided in symptomatic hypotensive patients (23).

### Administration and dosing of nitrate therapy

There is still uncertainty about the optimal combination treatment for acute heart failure upon hospital admission. Evidence from randomised controlled trials suggested that early administration of intravenous nitrates when combined with loop diuretics may provide improved outcomes. Patients who were not managed effectively in the early phase, i.e., first 6–12 h within presentation, have experienced poor outcomes (24). However, administration of continuous intravenous nitrate for more than 48 h led to greater attenuation of response compared with two intermittent 12-h infusions. In one study nitroglycerin doses had to be increased to maintain wedge pressure reduction at 12 h with an attenuated effect seen at 24 h. It has been suggested that concurrent use of angiotensin converting enzyme inhibitors may prevent nitrate tolerance and improve response to nitrates, given the involvement of angiotensin II in nitrate tolerance. Studies found that the use of angiotensin converting enzyme inhibitors preserved hemodynamic response and improved exercise tolerance, endothelial and left ventricular functions (23). Appropriate nitrate dose is important to achieve favourable hemodynamic effect and overcome tolerance (33). The use of low-dose nitroglycerin in acute heart failure may offer no or minimal clinical benefit (27, 38, 39), whereas higher doses provided remarkable benefits compared with standard therapy (27). Compared with standard of care, high dose of non-invasive (transdermal and sublingual) isosorbide dinitrate in addition to standard therapy within the first 48 h, was safe and greatly reduced natriuretic peptides. However, the benefit did not translate into improved mortality or rehospitalization rates (40). The relatively recent GALACTIC study that randomised patients with acute heart failure to either early intensive and sustained vasodilation or usual care did not demonstrate difference in mortality or hospitalization between groups (41). Another study (ELIZABETH) tested the efficacy of an early guideline-recommended care bundle in 75-year patients or older patients presenting with acute heart failure in the Emergency Department. The care bundle included early intravenous nitrate boluses in the first four hours, intravenous diuretics (moderate dose), and management of precipitating conditions such as atrial fibrillation, acute coronary syndrome, or infection. However, in comparison with the usual care (i.e., control group), the intervention did not significantly reduce the primary outcome (i.e., number of days alive and out of hospital at 30 days) (42).

## Clinical evidence

### Randomised controlled trials

Tables 3, 4 present the baseline characteristics, interventions, and outcomes of six identified randomised control trials that investigated nitrate therapy and were published after 1990 (1999–2008) (38, 45–47). The key inclusion criteria comprised pulmonary edema and decompensated heart failure. The cut-off systolic blood pressure measurement for exclusion was below 90–110 mmHg. Three trials excluded patients with acute myocardial infarction (38, 43, 44). When we pooled the data from the randomised trials, the recruited patients had a mean age of 62.9 years, 57.8% of patients were males, and 31.0% were smokers. The history of most common relevant comorbidities was reported for hypertension (63.0%), coronary artery disease (49.9%), myocardial infarction (47.6%), diabetes (44.4%), and heart failure that was reported in 53.0% of patients in only one study (43). The mean systolic and mean blood pressure measurements were 130.7 and 121.8 mmHg, respectively with a mean heart rate of 118.1 bpm. The mean cardiac index was 2.0 L/min/m^2^, PCWP was 28.1 mmHg, and ejection fraction was 40.3%. The nitrate therapy used as interventions were high-dose isosorbide dinitrate intravenous boluses and nitroglycerin intravenous infusion. The comparator groups varied between low-dose isosorbide dinitrate, milrinone, nesiritide, and furosemide combined with morphine. High-dose isosorbide dinitrate boluses were safe and effective in treating patients presenting with severe pulmonary edema (38, 45). Although nitroglycerin infusion was as effective as combined furosemide and morphine in acute pulmonary edema (43), it was not less effective than milrinone (44) or nesiritide (46, 47).

**Table 3:** Baseline characteristics of patients in nitrates randomised trials (after 1990).

Variable	Cotter et al. (45)	Sharon et al. (38)	Beltrame et al. (43)	Loh et al. (44)	VMAC trial. (46)	Chow et al. (47)	Pooled variable Mean ± SD or % (95% CI)
Recruitment years	–	Jan–June 1999	–	–	1999–2000	2006–2008	1999–2008
Key relevant criteria							
Inclusion	Pulmonary edema	Severe pulmonary edema	Pulmonary edema	Advanced DHF in ICU LVEF <45%; CI ≤2.5 L/min/m^2^; PCWP ≥18 mmHg	Decompensated CHF	Acute DHF	–
Exclusion	BP <110/70 mmHg	BP <110/70 mmHg STEMI	CS i.e., SBP ≤90 mmHg Overt acute myocardial infarction	Recent MI	SBP <90 mmHg Volume depletion CS	Incomplete data	–
Demographics							
Age (years)	–	73 ± 7	77 ± 6.6	55 ± 2	60 ± 14	72.3 ± 14.7	62.92 ± 10.10 (*n* = 290)
Male gender	26/52 (50%)	10/20 (50%)	14/32 (44%)	57/65 (88%)	86/143 (60%)	14/30 (47%)	57.86 (42.97–72.04) (207/342)
Smoking (active)	16/52 (16%)	6/20 (30%)	–	–	–	–	31.07 (21.10–42.01) (22/72)
Comorbidities							
Hypertension	28/52 (54%)	12/20 (60%)	18/32 (56%)	–	94/143 (66%)	23/30 (77%)	63.08 (57.15–68.72) (175/277)
Diabetes	20/52 (38%)	11/20 (55%)	12/32 (38%)	–	68/143 (48%)	12/30 (40%)	44.47 (38.58–50.48) (123/277)
CAD/IHD	31/52 (60%)	–	11/32 (34%)	–	90/143 (63%)	11/30 (37%)	49.95 (35.78–64.12) (143/257)
Myocardial infarction	–	12/20 (60%)	–	–	59/143 (41%)	–	47.66 (31.03–64.55) (71/163)
Heart failure	–	–	17/32 (53%)	–	–	–	17/32 (53%) One study
Vital signs/Hemodynamics							
SBP (mmHg)	–	–	161 ± 32	–	124 ± 23	–	130.76 ± 24.61 (*n* = 175)
MAP (mmHg)	132 ± 19	140 ± 16	–	–	–	92 ± 19	121.80 ± 18.42 (*n* = 102)
Heart rate (bpm)	117 ± 18	126 ± 15	115 ± 21	–	–	–	118.11 ± 18.35 (*n* = 104)
Respiratory rate (bpm)	42 ± 17	40 ± 5	32 ± 6	–	–	–	38.53 ± 11.36 (*n* = 104)
Cardiac index (L/min/m^2^)	–	–		1.8 ± 0.4	2.1 ± 0.8	–	2.0 ± 0.67 (*n* = 208)
PCWP	–	–		28.6 ± 6.5	28 ± 5.7	–	28.18 ± 5.94 (*n* = 208)
Ejection fraction (%)	42.3 ± 11	43 ± 6	40 ± 14 (n = 69)	–	–	36 ± 17	40.32 ± 12.32 (*n* = 171)
Laboratory tests							
Serum creatinine	–	–	–	–	–	1.3 ± 0.4 mg/dl 114.92 ± 35.36 mmol/L	114.92 ± 35.36 (*n* = 30)
GFR (ml/min)	–	–	–	–	–	52.5 ± 25.5	

95% CI, confidence interval; bpm, beats or breaths per minute; CAD, coronary artery disease; CI, cardiac index; CHF, congestive heart failure; CS, cardiogenic shock; DHF, decompensated heart failure; GFR, glomerular filtration rate; ICU, intensive care unit; IHD, ischemic heart disease; LVEF, left ventricular ejection fraction; MAP, mean arterial pressure; min, minute(s); PCWP, pulmonary capillary wedge pressure; SBP, systolic blood pressure; SD, standard deviation; STEMI, ST-segment elevation myocardial infarction; M, milrinone; NE, norepinephrine; PAC, pulmonary artery catheter; PCI, percutaneous coronary intervention; RBC, red blood cell; RRT, renal replacement therapy; SOFA, Sequential Organ Failure Assessment; TD, thermodilution.

**Table 4:** Interventions and outcomes reported in nitrates randomised trials (after 1990).

Variable	Cotter et al. (45)	Sharon et al. (38)	Beltrame (43)	Loh et al. (44)	VMAC trial (46)	Chow et al. (47)
Nitrate group size	52/110	20/40 (high-dose ISDN)	37/69	65/125	143/489	44/89
Intervention(s)						
Intervention group	ISDN 3 mg IV q5min (high dose)	ISDN IV boluses of 4 mg q4min	NTG/NAC	NTG for 24 hr to 5 days	NTG determined per investigator discretion	NTG for 48 h 10 μg/min titrated q5 min for 48 h, or until symptomrelief, SBP ≤90 mmHg, or max rate 200 μg/min
			NTG 2.5 μg/min with NAC at 6.6 μg/min over 24 h NTG infusion rate could be increased to 5 μg/min after 15 min and/or 10 μg/min at 60 min according to clinical response			
				NTG 5–25 μg/min with dose titration at rate of 10–25 μg/min every 3–5 min until one of the following: rate of 200 μg/min, 20 mmHg reduction in MAP, decrease in MAP (i.e., ≤ 55 mmHg), or NTG-related significant adverse event		
Comparator	ISDN 1 mg/h increased by 1 mg/h q10 min Furosemide 80 mg IV q15min	BiPAP ventilation ISDN 10 μmol/min increased q5–10 min by 10 μmol/min	Furosemide 40 mg IV bolus as de novo therapy ortwice patient's previous daily maintenancedose An equivalent second dose was administered at 60min according to response Morphine IV 1–2 mg/5 min to max dose of 10 mg	Milrinone for 24 h to 5 days 50 μg/kg over 10 min, then 0.50 μg/kg/min	Nesiritide Fixed-dose: 2-µg/kg, then 0.01 µg/kg/min Adjustable dose: infusion can be increased q3hr to max of 0.03 µg/kg/min	Nesiritide 2 mg/kg, then 0.01 mg/kg/min for atleast 48 h
Comparator 2	–	–	–	–	Placebo	–
Both received	Oxygen 10 L Furosemide 40 mg IV Morphine 3 mg IV	Oxygen 10 L Furosemide 80 mg IV Morphine 3 mg IV	No additional therapy was used unless needed	Dobutamine was added as per protocol	–	Standard therapy: diuretic, BB, ACEI/ARB, spironolactone
Outcomes (Intervention vs comparator)						
Surrogates						
Reduction in BP	MAP: by 19% vs 15%; *P *= 0.26	–	–	–	–	–
Reaching HD goal	–	–	–	8/59 (14%) vs 26/58 (45%); *P *< 0.0005 At 24 h: *P *= 0.026	–	–
Others	–	Significant improvement in O_2_%, RR, HR with ISDN	Significant improvement in RR, HR, systolic BP in both groups	–	Δ in CI and PCWP at 3 h: CI: 0.2 vs 0.1 PCWP: -3.8 vs -5.8	No difference in cytokines or renal parameters
Vasoactive agents use	–	–	–	52/65 (80%) vs 12/60 (20%)^a^	21/216 (9.7%) vs 27/273 (9.9%); NS	–
Agent(s) used				Dobutamine	Dobutamine Dopamine	–
Clinical						
Need for MV	7/52 (13%) vs 21/52 (40%); *P *= 0.0041	16/20 (80%) vs 4/20 (20%); *P *= 0.0004	–	–	–	–
Death	1/52 (2%) vs 3/52 (6%); *P *= 0.61	0 vs 2/20 (10%); *P *= 0.49	No difference between groups	–	–	–
Death and MV need	–	5/20 (25%) vs 17/20 (85%); *P *= 0.0003 Within 24 hr	–	–	–	–
In-hospital MI	9/52 (17%) vs 19/52 (37%); *P *= 0.047	11/20 (55%) vs 2/20 (10%); *P *= 0.006	6/37 (16%) vs 4/32 (12%)	–	Angina: 5/216 (2%) vs 5/273 (2%); *P *= 0.76	–
LOS (hospital)	–	–	5.6 ± 3 days (all patients); NS between groups	–	–	–
Conclusion	In patients with severe pulmonary edema, high-dose ISDN (i.e., repeated boluses after low-dose IV) was effective and safe. High-dose was more effective than low-dose.	In patients with severe pulmonary edema, high-dose ISDN was better and safer than BiPAP plus conventional therapy.	In patients with acute pulmonary edema, NTG/NAC was of comparable efficacy to furosemide/morphine.	In patients with DHF, milrinone was more effective than IV NTG in attaining and maintaining improved HD.	In hospitalised patients with decompensated CHF, nesiritide was more effective than IV NTG or control in improving symptoms and HD.	Combination of two agents of different mode of action may offer HD advantages compared with monotherapy.

Δ, change; ACEI, angiotensin-converting enzyme inhibitors; ARBs, angiotensin II receptor antagonists, BB, beta-blockers; BiPAP, bilevel positive airway ventilation; CHF, congestive heart failure; CI, cardiac index; h, hour(s); DHF, decompensated heart failure; HD, hemodynamic(S); HR, heart rate; ISDN, isosorbide dinitrate; IV, intravenous; L, liter; LOS, length of stay; MAP, mean arterial blood pressure; max, maximum; MI, myocardial infarction; min, minute(s); MV, mechanical ventilation; NAC, N-acetylcysteine; NS, non-significant; NTG, nitroglycerine; O_2_%, oxygen saturation; PCWP, pulmonary capillary wedge pressure; q, every; RR, respiratory rate; SBP, systolic blood pressure.

^a^ 12/60 (20%) received milrinone plus NTG, and 2/60 (3%) received milrinone plus both dobutamine and NTG.

### Observational studies

Seven non-randomised studies that investigated nitrate therapy and were published after 1990 (2001–2014) are described in Tables 5, 6 (24, 34, 39, 50, 48, 51). Patients presented with acute heart failure or CS with pulmonary edema. One study excluded patients with acute myocardial infarction (48), and another if myocardial infarction required immediate intubation (39). Three studies specified systolic blood pressure above 110 mmHg (24, 34, 49), and one study specified a cut-off of 160 mmHg or above (39). Our pooled data yielded a mean age of the enrolled patients of 74.0 years, 45.6% of patients were males, and 49.1% were smokers. The frequently reported comorbidities were hypertension (83.1%), heart failure (67.3%), diabetes (42.3%), dyslipidemia (41.4%), coronary artery disease (39.0%), renal impairment (29.9%), stroke (15.0%), and myocardial infarction (24.1%) that was report in one study (39). The mean systolic and diastolic blood pressure measurements were 157.3 and 78.9 mmHg, respectively, with a mean heart rate of 92.5 bpm. The mean lactate level was 1.53 mmol/L. The nitrate therapy used were interventions isosorbide dinitrate and nitroglycerin. The comparator groups varied between different nitrate doses, diuretics, or control. The addition of nitrate bolus to nitrate infusion was not associated with increased hypotensive episodes. High-dose nitroglycerin was associated with more frequent intubation and intensive care unit admission than lower dose without excess in adverse events. Although early administration of nitrate along with diuretics reduced the length of stay, there was not benefit in reducing the risk of mortality.

**Table 5:** Baseline characteristics of patients in nitrates non- randomised trials (after 1990).

Variable	Freund et al. (50)	Lemachatti et al. (49)	Costanzo et al. (48)	Levy et al. (39)	Aziz et al. (51)	Herrero-Puente et al. (34)	Mohan et al. (24)	Pooled variable
								Mean ± SD or% (95% CI)
Recruitment years	Jan–December 2007 (case series)	2007 (before) 2014 (after)	2001–2004	–	Over 12 months	May 2009 Nov–December 2011	Jan–August 2013	2001–2014
Key relevant criteria								
Inclusion	CHF; Age ≥75	CS acute pulmonary edema; Age ≥75 (SBP >110 mmHg, signs of pulmonary oedema, no shock and no hypoperfusion)	Acute DHF failure	Severe DHF HTN (SBP ≥160 mmHg or MAP ≥120 mmHg, refractory to initial therapy	Acute DHF	Acute heart failure SBP >110 mmHg	Acute heart failure per guidelines (SBP > 110 mmHg, pulmonary congestion, no severe aortic/pulmonary stenosis)	–
Exclusion	Inconsistent medical charts	Incomplete records	Acute myocardial infarction	STEMI Need for immediate intubation	–	–	–	–
Demographics								
Age (years)	86.6 ± 5.8	85 ± 6	70.2 ± 14.1	61.4 ± 14.8	72 ± 13	78.6 ± 10.2	77 ± 11	74.05 ± 12.16 (*n* = 2,758)
Male gender	–	–	1,791/3,947 (45.4%)	19/29 (65.5%)	20/46 (43%)	340/796 (42.7%)	22/40 (55%)	45.65 (41.87–49.45) (2,192/4,858)
Smoking (active)	9/25 (36%)	77/232 (33%)	–	14/29 (48.3%)	36/46 (80%)	–	–	49.13 (27.19–71.25) (136/332)
Comorbidities								
Hypertension	18/25 (72%)	201/232 (87%)	–	26/29 (89.7%)	37/46 (82%)	686/796 (86.2%)	29/40 (73%)	83.11 (78.47–87.28) (997/1,168)
Diabetes	–	81/232 (35%)	–	–	24/46 (53%)	378/796 (47.5%)	14/40 (35%)	42.39 (33.83–51.19) (497/1,114)
Dyslipidemia	–	–	–	–	15/46 (32%)	371/796 (46.6%)	–	41.45 (28.84–54.64) (386/842)
CVA/TIA	–	30/232 (13%)	–	–	–	122/796 (15.3%)	8/40 (20%)	15.05 (12.96–17.33) (160/1,068)
Coronary artery disease/IHD	–	88/232 (38%)	–	11/29 (37.9%)	14/46 (31%)	315/796 (39.6%)	18/40 (45%)	39.05 (36.22–41.94) (446/1,143)
Myocardial infarction	–	–	–	7/29 (24.1%)	–	–	–	7/29 (24.1%)
Heart failure	7/25 (28%)	160/232 (69%)	–	26/29 (89.7%)	33/46 (73%)	550/796 (69.1%)	–	67.37 (57.33–76.66) (776/1,128)
Renal impairment	–	–	–	6/29 (20.7%) dialysis	–	190/796 (23.9%)	19/40 (47%)	29.99 (16.84–45.09) (215/865)
Vital signs/Hemodynamics								
Systolic BP (mmHg)	160 ± 25	138 ± 26	162 ± 37	–	150 ± 21	155.5 ± 31.8	131 ± 27	157.38 ± 34.03 (*n* = 2,729)
Diastolic BP (mmHg)	90 ± 22	77 ± 17	–	–	87 ± 11	–	74 ± 18	78.93 ± 16.67 (*n* = 343)
MAP (mmHg)	–	–	–	157.2 ± 25.7	–	–	–	157.2 ± 25.7 (*n* = 29)
Heart rate (bpm)	96 ± 23	84 ± 19	93 ± 23	116.3 ± 25.5	–	93 ± 25.3	95 ± 28	92.53 ± 23.43 (*n* = 2,712)
Respiratory rate (bpm)	30 ± 7	26 ± 56	–	31.1 ± 6.7	–	24.1 ± 7.4	–	24.83 ± 17.78 (*n* = 1,082)
Pulse oximetry (%)	92 (87–95)^a^	95 (90–97)^a^	–	92.7 ± 5.4	–	–	–	93.85 ± 1.67 (*n* = 286)
Ejection fraction (%)	–	–	–	35.7 (28.7–42.7) Mean: 35.7 ± 4.04	32 ± 16	–	–	33.43 ± 11.41 (*n* = 75)
Laboratory tests								
Lactate (mmol/L)	1.8 (1.3–2.6)^a^	1.5 ± 0.8	–	–	–	–	–	1.53 ± 0.76 (*n* = 257)
pH	7.4 ± 0.07	–	–	–	–	–	–	
Bicarbonate (mmol/L)	–	24 (22–27)^a^	–	–	–	–	–	
Hemoglobin (g/dl)	–	11.8 ± 1.8	12.5 ± 2.2	–	–	–	–	12.41 ± 2.14 (*n* = 1,822)
Troponin (ng/ml)	–	–	–	–	0.24 ± 0.46	–	–	0.24 ± 0.46 (*n* = 46)
BNP (pg/ml)	–	–	1,389 ± 2,179	1,781.6 ± 2,683.0	1,494 ± 1,043	–	–	1,398.7 ± 2,156.7 (*n* = 1,665)
BUN (mg/dl)	–	–	27.5 ± 16	–	–	–	–	27.5 ± 16 (*n* = 1,590)
Serum creatinine (mmol/L)	94 (74–116)^a^	99 (75–127)^a^	141.44 ± 97.24	309.4 ± 167.96	150.28 ± 132.6	–	–	138.57 ± 85.88 (*n* = 1,922)
GFR (ml/min)	–	–	–	–	42 ± 17	–	–	

BP, blood pressure; bpm, beats or breaths per minute; BNP, brain natriuretic peptide; BUN, blood urea nitrogen; CHF, congestive heart failure; CS, cardiogenic shock; CVA, cerebrovascular accident; DHF, decompensated heart failure; GFR, glomerular filtration rate; HTN, hypertension; IHD, ischemic heart disease; MAP, mean arterial blood pressure; MD, mean difference; SD, standard deviation; STEMI, ST-segment elevation myocardial infarction; SBP, systolic blood pressure; TIA, transient ischemic attack.

^a^ Converted to mean ± standard deviation

**Table 6:** Interventions and outcomes reported in nitrates non- randomised trials (after 1990).

Variable	Freund et al. (50)	Lemachatti et al. (49)	Costanzo et al. (48)	Levy et al. (39)	Aziz et al. (51)	Herrero-Puente et al. (34)	Mohan et al. (24)
Nitrate group size	25/136 (ISDN bolus)	2014: 232/368	1,590 (monotherapy)3,947 (with diuretic) *N* = 99,963	29/74	46/430	796/3,187	40/81
Intervention(s)							
Intervention group	ISDN bolus (3.8 ± 1.9 mg) ISDN infusion: 16/25 (71%)	ISDN use 2014 vs 2007: 97/232 (42%) vs 25/136 (18%) (*P *< 0.01) ISDN bolus dose: 5 mg vs 3 mg (*P *< 0.01) Odds ratio to receive nitrate in 2014: 3.94 (2.22–7.01)	NTG NTG and diuretic	High dose NTG Mean bolus dose: 6.5 mg Initial rate: 23.6 Final rate: 50.2 μg/min	NTG infusion and diuretics	NTG infusion 5–200 mg/min for 6–48 h	Nitrates as per Guidelines recommendations
Comparator 1	No bolus ISDN ISDN infusion: 18/111 (16%)	Before and after design	Diuretic	Non-intervention Initial NTG rate: 31.7 μg/min Final NTG rate: not available	Furosemide	Control	–
Comparator 2	–	–	–	–	Neither diuretics nor NTG	–	–
Both received	–	–	–	Initially, all patients began with NTG 0.3–0.5 μg/kg/min Titration: 20 μg/min q1–3 min Max: fixed at 400 μg/min	–	–	–
Outcomes							
Surrogates							
Reduction in BP	Post treatment 116 ± 19 vs 116 ± 18 (*P *= 0.99)	–	–	Δ MAP -50.0 mmHg	–	–	–
Others	–	–	–	Δ Pulse rate -18 bpm Δ RR -8.0 bpm Δ SpO_2_ 3.0%	–	–	–
Clinical							
Intubation	–	–	–	13.8% vs 26.7%	–	–	–
Death	1/25 (4%) vs 11/111 (10%) (*P *= 0.3)	19/232 (8%) vs 15/136 (11%) (*P *= 0.5)	NTG with diuretic vs diuretic 2.8% vs 3.2% (*P *= 0.19)	–	4/46 (8%) vs 11/127 (9%) vs 42/257 (16%) (*P *= 0.039)	3-day: 24/796 (3%) vs 66/2,382 (2.8%) (*P *= 0.72) 7-day: 41/796 (5.2%) vs 107/2,382 (4.5%) (*P *= 0.45) 14-day: 60/796 (7.5%) vs 164/2,382 (6.9%) (*P *= 0.53)	–
Death and ICU admission	–	44/232 (19%) vs 36/136 (27%) (*P *= 0.1)	–	–	–	–	–
Hospital readmission	–		–	–	22/46 (48%) vs 68/127 (53%) vs 143/257 (56%) (NS)	–	–
Survival	–		–	–	Survival probability 87% vs 82% vs 79% (*P *= 0.0001)	–	–
Cardiovascular complications	–		–	20.7% vs 289%	–	–	–
30-day mortality	–	–	–	–	–	84/796 (10.6%) vs 224/2,382 (9.4%) (*P *= 0.34)	–
LOS (hospital)	14 vs 11 days (*P *= 0.2)	10 (5–16) vs 10 (5–15) (*P *= 0.9) days	–	4.1 vs 6.2 days	6 ± 4 vs 9 ± 12 vs 8 ± 8 (*P *= 0.01)	–	–
Hospital readmission	–	From ED to admission 213/232 (92%) vs 122/136 (90%) (*P *= 0.5)	–	–	At 30-day 5/46 (13%) vs 18/127 (14%) vs 33/257 (13%) (NS)	From ED to admission 657/796 (82.5%) vs 1,829/2,382 (76.8%) (*P *= 0.001)	–
ICU admission	7/25 (28%) vs 12/111 (11%) (*P* = 0.04)	30/232 (13%) vs 23/136 (17%) (*P *= 0.3)	–	37.9% vs 80.0%	–	–	–
New ED visit at 30-day	–	–	–	DHF: 27.6% vs 22.2%	–	At 30-day 117/796 (17.3%) vs 348/2,382 (16.9%) (*P *= 0.81)	–
Conclusion	ISDN did not cause more hypotension with bolus use.	In elderly patients with acute heart failure and treated with ISDN boluses, there was no significant benefit in outcomes.	Vasodilators did not increase inpatient mortality when compared with alternative regimens.	Intubation, BiPAP, and ICU admission occurred less frequently than anticipated with high-dose NTG.	In patients with acute DHF admitted to ED, early NTG as add-on to diuretics, reduced LOS with a trend towards lower composite of mortality and acute DHF readmission.	In patients with acute heart failure, IV nitrates did not reduce mortality.	Only 12% of patients who are suitable candidate for IV nitrates received it.

Δ, change; BiPAP, bilevel positive airway ventilation; bpm, beats or breaths per minute; DHF, decompensated heart failure; ED, emergency department; h, hour(s); ICU, intensive care unit; ISDN, isosorbide dinitrate; IV, intravenous(ly); LOS, length of stay; MAP, mean arterial pressure; min, minute(s); NTG, nitroglycerin; NS, not significant; RR, respiratory rate; SpO_2_, oxygen saturation.

### Overall characteristics of three study types

Overall, the pooled variables of the three study types showed noticeable variations in patients’ characteristics (Tables 2, 3, 5).

## Current position and future direction

Currently, there is no recommendation that favors a therapeutic regimen according to nitrate therapy vs. usual care (22). In myocardial infarction, intravenous nitrates are usually used for 24–48 h in patients presenting with large anterior myocardial infarction, acute myocardial infarction with congestive heart failure, and ongoing ischemia or hypertension. The infusion can be continued beyond 48 h in the presence of ongoing pulmonary congestion or recurrent angina (52, 53). Nitrates should be avoided if systolic blood pressure is below 90 mmHg, in the presence of significant bradycardia (i.e., heart rate below 50 bpm) or tachycardia, or in patients with right ventricular infarction (52). In the absence of hypotension, intravenous nitrates may be given as an adjunctive to diuretic agents in patients with decompensated heart failure (54, 55). Intravenous nitrate is administered to relieve the symptoms of acute heart failure when systolic blood pressure is above 110 mmHg in the absence of severe aortic or mitral stenosis (22, 56). The infusion is usually set to start at low rate then can be up-titrated according to clinical status and blood pressure measurements (22). An initial nitrate bolus may precede the continuous infusion. Moreover, repeated boluses may be considered as well, for example, 1–2 mg nitroglycerin boluses in patients with severe hypertension and acute pulmonary edema (22). Although intravenous nitrates seem to be most effective in acute heart failure patients with hypertension or myocardial ischemia, it is unknown whether this translates to their use in daily practice as the real-world data is not yet clearly defined (24). Other vasodilators may be considered. When nitroglycerin was compared with milrinone (44) and nesiritide (46) in patients with acute decompensated heart failure (Table 4), both agents were more effective than nitroglycerin in improving hemodynamic parameters. Moreover, other potentially effective vasodilator agents in acute heart failure include intravenous enalaprilat, nicardipine, or nitroprusside due to the reduction of preload, afterload, or both, respectively. However, none of these agents were compared with intravenous nitrates. In the absence of hypotension, the authors commonly use intravenous nitrates as first-line therapy in daily clinical practice to relieve chest pain secondary to acute coronary syndrome, acute heart failure, and pulmonary edema. The use of intravenous nitrate therapy in the pre-shock state or SCAI stage B with its range of presentations (i.e., pulmonary edema, heart failure either *de novo* or acute-on-chronic, or myocardial infarction) can be considered an extrapolation from the available evidence that only demonstrated favourable hemodynamic effects without a confirmed mortality benefit. Although a novel therapy could address the limitations of nitrate therapy, more is anticipated and needed to re-establish the role of nitrates within the contemporary context given the anticipated burden of shock on healthcare sector. There is unmet need to reassess the benefit of intravenous nitrate therapy after the introduction of SCAI shock stage classification in well-designed prospective studies.

## Conclusion

Patients in pre-shock state may present with pulmonary edema, acute heart failure, or myocardial infarction complicated with CS. Nitrate therapy is considered a traditional treatment that improves hemodynamic parameters and reduces dyspnea, congestion, and pain. However, there is no robust evidence to confirm benefit in terms of mortality outcomes and the uncertainty continues with the introduction of the contemporary shock stages classification.

## References

[1] Zweck E Thayer KL Helgestad OKL Kanwar M Ayouty M Garan AR Phenotyping cardiogenic shock. J Am Heart Assoc. (2021) 10(14):e020085. 10.1161/JAHA.120.020085 34227396 PMC8483502

[2] Baran DA Grines CL Bailey S Burkhoff D Hall SA Henry TD SCAI Clinical expert consensus statement on the classification of cardiogenic shock: this document was endorsed by the American college of cardiology (ACC), the American Heart Association (AHA), the Society of Critical Care Medicine (SCCM), and the Society of Thoracic Surgeons (STS) in April 2019. Catheter Cardiovasc Interv. (2019) 94(1):29–37. 10.1002/ccd.28329 31104355

[3] van Diepen S. Norepinephrine as a first-line inopressor in cardiogenic shock: oversimplification or best practice? J Am Coll Cardiol. (2018) 72(2):183–6. 10.1016/j.jacc.2018.04.052 29976292

[4] Kaddoura R Elmoheen A Badawy E Eltawagny MF Seif MA Bashir K Vasoactive pharmacologic therapy in cardiogenic shock: a critical review. J Drug Assess. (2021) 10(1):68–85. 10.1080/21556660.2021.1930548 34350058 PMC8293961

[5] Naidu SS Baran DA Jentzer JC Hollenberg SM van Diepen S Basir MB SCAI SHOCK stage classification expert consensus update: a review and incorporation of validation studies: this statement was endorsed by the American college of cardiology (ACC), American college of emergency physicians (ACEP), American Heart Association (AHA), European Society of Cardiology (ESC) Association for Acute Cardiovascular Care (ACVC), International Society for Heart and Lung Transplantation (ISHLT), Society of Critical Care Medicine (SCCM), and Society of Thoracic Surgeons (STS) in December 2021. J Am Coll Cardiol. (2022) 79(9):933–46. 10.1016/j.jacc.2022.01.018 35115207

[6] Held P. Effects of nitrates on mortality in acute myocardial infarction and in heart failure. Br J Clin Pharmacol. (1992) 34 Suppl 1(Suppl 1):25S–8S. 10.1111/j.1365-2125.1992.tb04145.x 1633075 PMC1381219

[7] Hollenberg SM. Vasodilators in acute heart failure. Heart Fail Rev. (2007) 12(2):143–7. 10.1007/s10741-007-9017-2 17447137

[8] Kaddoura R Elbdri S. Current evidence in the diagnosis and management of cardiogenic shock complicating acute coronary syndrome. Rev Cardiovasc Med. (2021) 22(3):691–715. 10.31083/j.rcm2203078 34565070

[9] Baran DA Long A Badiye AP Stelling K. Prospective validation of the SCAI shock classification: single center analysis. Catheter Cardiovasc Interv. (2020) 96(7):1339–47. 10.1002/ccd.29319 33026155 PMC7821022

[10] Hanson ID Tagami T Mando R Kara Balla A Dixon SR Timmis S SCAI Shock classification in acute myocardial infarction: insights from the national cardiogenic shock initiative. Catheter Cardiovasc Interv. (2020) 96(6):1137–42. 10.1002/ccd.29139 32672388

[11] Jentzer JC van Diepen S Barsness GW Henry TD Menon V Rihal CS Cardiogenic shock classification to predict mortality in the cardiac intensive care unit. J Am Coll Cardiol. (2019) 74(17):2117–28. 10.1016/j.jacc.2019.07.077 31548097

[12] Jentzer JC Baran DA van Diepen S Barsness GW Henry TD Naidu SS Admission society for cardiovascular angiography and intervention shock stage stratifies post-discharge mortality risk in cardiac intensive care unit patients. Am Heart J. (2020) 219:37–46. 10.1016/j.ahj.2019.10.012 31710843

[13] Lawler PR Berg DD Park JG Katz JN Baird-Zars VM Barsness GW The range of cardiogenic shock survival by clinical stage: data from the critical care cardiology trials network registry. Crit Care Med. (2021) 49(8):1293–302. 10.1097/CCM.0000000000004948 33861557

[14] Pareek N Dworakowski R Webb I Barash J Emezu G Melikian N SCAI Cardiogenic shock classification after out of hospital cardiac arrest and association with outcome. Catheter Cardiovasc Interv. (2021) 97(3):E288–97. 10.1002/ccd.28984 32445610

[15] Schrage B Dabboura S Yan I Hilal R Neumann JT Sörensen NA Application of the SCAI classification in a cohort of patients with cardiogenic shock. Catheter Cardiovasc Interv. (2020) 96(3):E213–9. 10.1002/ccd.28707 31925996

[16] Thayer KL Zweck E Ayouty M Garan AR Hernandez-Montfort J Mahr C Invasive hemodynamic assessment and classification of in-hospital mortality risk among patients with cardiogenic shock. Circ Heart Fail. (2020) 13(9):e007099. 10.1161/CIRCHEARTFAILURE.120.007099 32900234 PMC10243474

[17] Jentzer JC Schrage B Holmes DR Dabboura S Anavekar NS Kirchhof P Influence of age and shock severity on short-term survival in patients with cardiogenic shock. Eur Heart J Acute Cardiovasc Care. (2021) 10(6):604–12. 10.1093/ehjacc/zuaa035 33580778

[18] Jentzer JC Burstein B Van Diepen S Murphy J Holmes DR Jr Bell MR Defining shock and preshock for mortality risk stratification in cardiac intensive care unit patients. Circ Heart Fail. (2021) 14(1):e007678. 10.1161/CIRCHEARTFAILURE.120.007678 33464952

[19] Tarvasmäki T Harjola VP Nieminen MS Siirilä-Waris K Tolonen J Tolppanen H Acute heart failure with and without concomitant acute coronary syndromes: patient characteristics, management, and survival. J Card Fail. (2014) 20(10):723–30. 10.1016/j.cardfail.2014.07.008 25079300

[20] Hollenberg SM Warner Stevenson L Ahmad T Amin VJ Bozkurt B Butler J 2019 ACC expert consensus decision pathway on risk assessment, management, and clinical trajectory of patients hospitalized with heart failure: a report of the American College of Cardiology Solution Set Oversight Committee [published correction appears in J Am Coll Cardiol. 2020 Jan 7;75(1):132]. J Am Coll Cardiol. (2019) 74(15):1966–2011. 10.1016/j.jacc.2019.08.001 31526538

[21] Hernandez-Montfort J Sinha SS Thayer KL Whitehead EH Pahuja M Garan AR Clinical outcomes associated with acute mechanical circulatory support utilization in heart failure related cardiogenic shock. Circ Heart Fail. (2021) 14(5):e007924. 10.1161/CIRCHEARTFAILURE.120.007924 33905259

[22] McDonagh TA Metra M Adamo M 2021 ESC guidelines for the diagnosis and treatment of acute and chronic heart failure. Eur Heart J. (2021) 42(36):3599–726. 10.1093/eurheartj/ehab368 34447992

[23] Piper S McDonagh T. The role of intravenous vasodilators in acute heart failure management. Eur J Heart Fail. (2014) 16(8):827–34. 10.1002/ejhf.123 25100108

[24] Mohan M Hawkey S Baig F Choy AM Lang CC. Underutilization of IV nitrates in the treatment of acute heart failure. Cardiovasc Ther. (2015) 33(4):247–52. 10.1111/1755-5922.12135 25981786

[25] Thadani U Ripley TL. Side effects of using nitrates to treat heart failure and the acute coronary syndromes, unstable angina and acute myocardial infarction. Expert Opin Drug Saf. (2007) 6(4):385–96. 10.1517/14740338.6.4.385 17688382

[26] den Uil CA Brugts JJ. Impact of intravenous nitroglycerin in the management of acute decompensated heart failure. Curr Heart Fail Rep. (2015) 12(1):87–93. 10.1007/s11897-014-0230-8 25301529

[27] Alzahri MS Rohra A Peacock WF. Nitrates as a treatment of acute heart failure. Card Fail Rev. (2016) 2(1):51–5. 10.15420/cfr.2016:3:3 28785453 PMC5490950

[28] Thadani U. Secondary preventive potential of nitrates in ischaemic heart disease. Eur Heart J. (1996) 17 Suppl F:30–6. 10.1093/eurheartj/17.suppl_F.30 8960445

[29] Yusuf S Collins R MacMahon S Peto R. Effect of intravenous nitrates on mortality in acute myocardial infarction: an overview of the randomised trials. Lancet. (1988) 1(8594):1088–92. 10.1016/S0140-6736(88)91906-X 2896919

[30] ISIS-4: a randomised factorial trial assessing early oral captopril, oral mononitrate, and intravenous magnesium sulphate in 58,050 patients with suspected acute myocardial infarction. ISIS-4 (fourth international study of infarct survival) collaborative group. Lancet. (1995) 345(8951):669–85. 10.1016/S0140-6736(95)90865-X 7661937

[31] Six-month effects of early treatment with lisinopril and transdermal glyceryl trinitrate singly and together withdrawn six weeks after acute myocardial infarction: the GISSI-3 trial. Gruppo italiano per lo studio della sopravvivenza nell'Infarto miocardico. J Am Coll Cardiol. (1996) 27(2):337–44. 8557903

[32] Wakai A McCabe A Kidney R Brooks SC Seupaul RA Diercks DB Nitrates for acute heart failure syndromes. Cochrane Database Syst Rev. (2013) 2013(8):CD005151. 23922186 10.1002/14651858.CD005151.pub2 PMC8101690

[33] Elkayam U Bitar F Akhter MW Khan S Patrus S Derakhshani M. Intravenous nitroglycerin in the treatment of decompensated heart failure: potential benefits and limitations. J Cardiovasc Pharmacol Ther. (2004) 9(4):227–41. 10.1177/107424840400900403 15678242

[34] Herrero-Puente P Jacob J Martín-Sánchez FJ Vázquez-Álvarez J Martínez-Camblor P Miró Ó Influence of intravenous nitrate treatment on early mortality among patients with acute heart failure. NITRO-EAHFE study. Rev Esp Cardiol (Engl Ed). (2015) 68(11):959–67. 10.1016/j.recesp.2014.12.017 25863419

[35] Gray A Goodacre S Seah M Tilley S. Diuretic, opiate and nitrate use in severe acidotic acute cardiogenic pulmonary oedema: analysis from the 3CPO trial. QJM. (2010) 103(8):573–81. (Abstract only). 10.1093/qjmed/hcq077 20511258

[36] Tarvasmäki T Harjola VP Tolonen J Siirilä-Waris K Nieminen MS Lassus J. Management of acute heart failure and the effect of systolic blood pressure on the use of intravenous therapies. Eur Heart J Acute Cardiovasc Care. (2013) 2(3):219–25. 10.1177/2048872613492440 24222833 PMC3821822

[37] Carr A De Iorio F Cowie MR. Variability in use of IV nitrates and diuretics in acute HF: a virtual patient clinical decision-making study. Br J Cardiol. (2018). 10.5837/bjc.2018.002. Available at: Variability in use of IV nitrates and diuretics in acute HF: a ‘virtual patient’ clinical decision-making study—The British Journal of Cardiology (bjcardio.co.uk)—(Accessed August 31, 2022).

[38] Sharon A Shpirer I Kaluski E Moshkovitz Y Milovanov O Polak R High-dose intravenous isosorbide-dinitrate is safer and better than Bi-PAP ventilation combined with conventional treatment for severe pulmonary edema. J Am Coll Cardiol. (2000) 36(3):832–7. 10.1016/S0735-1097(00)00785-3 10987607

[39] Levy P Compton S Welch R Delgado G Jennett A Penugonda N Treatment of severe decompensated heart failure with high-dose intravenous nitroglycerin: a feasibility and outcome analysis. Ann Emerg Med. (2007) 50(2):144–52. 10.1016/j.annemergmed.2007.02.022 17509731

[40] Breidthardt T Noveanu M Potocki M Reichlin T Egli P Hartwiger S Impact of a high-dose nitrate strategy on cardiac stress in acute heart failure: a pilot study. J Intern Med. (2010) 267(3):322–30. 10.1111/j.1365-2796.2009.02146.x 19694900

[41] Kozhuharov N Goudev A Flores D Maeder MT Walter J Shrestha S Effect of a strategy of comprehensive vasodilation vs usual care on mortality and heart failure rehospitalization among patients with acute heart failure: the GALACTIC randomized clinical trial. JAMA. (2019) 322(23):2292–302. 10.1001/jama.2019.18598 31846016 PMC6990838

[42] Freund Y Cachanado M Delannoy Q Laribi S Yordanov Y Gorlicki J Effect of an emergency department care bundle on 30-day hospital discharge and survival among elderly patients with acute heart failure: the ELISABETH randomized clinical trial. JAMA. (2020) 324(19):1948–56. 10.1001/jama.2020.19378 33201202 PMC7672513

[43] Beltrame JF Zeitz CJ Unger SA Brennan RJ Hunt A Moran JL Nitrate therapy is an alternative to furosemide/morphine therapy in the management of acute cardiogenic pulmonary edema. J Card Fail. (1998) 4(4):271–9. 10.1016/S1071-9164(98)90232-9 9924848

[44] Loh E Elkayam U Cody R Bristow M Jaski B Colucci WS. A randomized multicenter study comparing the efficacy and safety of intravenous milrinone and intravenous nitroglycerin in patients with advanced heart failure. J Card Fail. (2001) 7(2):114–21. 10.1054/jcaf.2001.24136 11420762

[45] Cotter G Metzkor E Kaluski E Faigenberg Z Miller R Simovitz A Randomised trial of high-dose isosorbide dinitrate plus low-dose furosemide versus high-dose furosemide plus low-dose isosorbide dinitrate in severe pulmonary oedema. Lancet. (1998) 351(9100):389–93. 10.1016/S0140-6736(97)08417-1 9482291

[46] Publication Committee for the VMAC Investigators (Vasodilatation in the Management of Acute CHF). Intravenous nesiritide vs nitroglycerin for treatment of decompensated congestive heart failure: a randomized controlled trial. JAMA. (2002) 287(12):1531–40. 10.1001/jama.287.12.1531 11911755

[47] Chow SL O’Barr SA Peng J Chew E Pak F Quist R Modulation of novel cardiorenal and inflammatory biomarkers by intravenous nitroglycerin and nesiritide in acute decompensated heart failure: an exploratory study. Circ Heart Fail. (2011) 4(4):450–5. 10.1161/CIRCHEARTFAILURE.110.958066 21576282

[48] Costanzo MR Johannes RS Pine M Gupta V Saltzberg M Hay J The safety of intravenous diuretics alone versus diuretics plus parenteral vasoactive therapies in hospitalized patients with acutely decompensated heart failure: a propensity score and instrumental variable analysis using the acutely decompensated heart failure national registry (ADHERE) database. Am Heart J. (2007) 154(2):267–77. 10.1016/j.ahj.2007.04.033 17643575

[49] Lemachatti N Philippon AL Bloom B Hausfater P Riou B Ray P Freund Y. Temporal trends in nitrate utilization for acute heart failure in elderly emergency patients: a single-centre observational study. Arch Cardiovasc Dis. (2016) 109(8–9):449–56. 10.1016/j.acvd.2016.01.014 27342805

[50] Freund Y Delerme S Boddaert J Baker E Riou B Ray P. Isosorbide dinitrate bolus for heart failure in elderly emergency patients: a retrospective study. Eur J Emerg Med. (2011) 18(5):272–5. 10.1097/MEJ.0b013e328345d72a 21499108

[51] Aziz EF Kukin M Javed F Pratap B Sabharwal MS Tormey D Effect of adding nitroglycerin to early diuretic therapy on the morbidity and mortality of patients with chronic kidney disease presenting with acute decompensated heart failure. Hosp Pract (1995). (2011) 39(1):126–32. (Abstract only). 10.3810/hp.2011.02.382 21441767

[52] Ryan TJ Antman EM Brooks NH Califf RM Hillis LD Hiratzka LF 1999 Update: aCC/AHA guidelines for the management of patients with acute myocardial infarction: executive summary and recommendations: a report of the American College of Cardiology/American Heart Association Task Force on practice guidelines (committee on management of acute myocardial infarction). Circulation. (1999) 100(9):1016–30. 10.1161/01.CIR.100.9.1016 10468535

[53] Aronow WS. Drug treatment of elderly patients with acute myocardial infarction: practical recommendations. Drugs Aging. (2001) 18(11):807–18. 10.2165/00002512-200118110-00002 11772121

[54] Yancy CW Jessup M Bozkurt B Butler J Casey DE Jr Drazner MH 2013 ACCF/AHA guideline for the management of heart failure: a report of the American college of cardiology foundation/American heart association task force on practice guidelines. J Am Coll Cardiol. (2013) 62(16):e147–239. 10.1016/j.jacc.2013.05.019 23747642

[55] Heidenreich PA Bozkurt B Aguilar D Allen LA Byun JJ Colvin MM 2022 AHA/ACC/HFSA guideline for the management of heart failure: a report of the American College of Cardiology/American Heart Association Joint Committee on Clinical Practice Guidelines. Circulation. (2022) 145(18):e895–e1032. 10.1161/CIR.0000000000001063 35363499

[56] McMurray JJ Adamopoulos S Anker SD Auricchio A Böhm M Dickstein K ESC Guidelines for the diagnosis and treatment of acute and chronic heart failure 2012: the task force for the diagnosis and treatment of acute and chronic heart failure 2012 of the European society of cardiology. Developed in collaboration with the Heart Failure Association (HFA) of the ESC [published correction appears in Eur J Heart Fail. 2013 Mar;15(3):361-2]. Eur J Heart Fail. (2012) 14(8):803–69. 10.1093/eurjhf/hfs105 22828712

